# Neurologic outcomes following intubation with non-depolarizing vs. depolarizing paralytics in status epilepticus: a target trial emulation

**DOI:** 10.62838/jccm-2026-0041

**Published:** 2026-10-30

**Authors:** Patrick King, Juan Garcia-Mendez, Aysun Tekin, Diksha Gowda, Anes Joguncic, Amos Lal

**Affiliations:** Department of Pulmonary and Critical Care Medicine, Mayo Clinic, Rochester, MN; Department of Anesthesiology, Mayo Clinic, Rochester, MN; Department of Epidemiology, Public Health Institute of Canton of Sarajevo

**Keywords:** status epilepticus, rapid sequence intubation, neuromuscular blockade, succinylcholine, rocuronium, target trial emulation, critical care

## Abstract

**Background:**

Significant controversy exists regarding the optimal pharmacologic approach for endotracheal intubation in patients suffering from status epilepticus (SE).

Study Aims: To evaluate post-hospitalization neurologic sequelae and other patient-centered outcomes in SE patients receiving neuromuscular blockers (NMBs). We hypothesized that ability to more rapidly reassess neurologic exam with depolarizing-NMBs (D-NMBs) would mediate improved neurologic outcomes compared to non-depolarizing-NMBs (ND-NMBs).

**Material and Methods:**

We analyzed a cohort of SE patients in a large, quaternary academic system from May 2000 to March 2024. Measured confounders were addressed using propensity score methods and inverse probability of treatment weighting. 480 qualifying adults across closely-affiliated health system hospitals undergoing their first intubation for suspected SE with documented paralytic choice were identified via proprietary institutional database using the key search phrase “status epilepticus” in emergency medicine notes. (Exposure: Paralytic selection during rapid sequence intubation – intervention (D-NMB) vs. control (ND-NMB)). As a surrogate towards functional neurologic outcomes, need for “escalation in level of care” at hospital discharge was assessed as primary outcome of interest. Secondary outcomes included major hyperkalemia-related events (ventricular or pulseless arrhythmias) and 30-day survival.

**Results:**

D-NMB use relative to ND-NMB use was associated with increased odds of escalation in level of care upon discharge (30.4% vs. 24.4%, odds ratio [OR] 1.35, 95% CI 1.01–1.81) in addition to decreased odds of home disposition upon hospital discharge (61.7% vs. 69.6%, OR 0.71, 95% CI 0.54–0.94). There was no difference in life-threatening peri-intubation cardiac events such as ventricular or pulseless arrhythmias (0.6% vs. 0.8%, p=0.64).

**Conclusions:**

Across 480 SE patients, ND-NMBs were associated with lower need for escalation in level of care upon hospital discharge compared to D-NMBs.

## Introduction

Status epilepticus (SE) is one of the most common neurologic emergencies encountered by acute care clinicians; the United States Nationwide Emergency Department Sample database identified at least 233,209 SE visits between 2010–2014 [1]. Patients with inadequate response to initial efforts to control seizures frequently require intubation in order to utilize general anesthetic infusions (e.g., propofol). Rapid sequence intubation (RSI) employs simultaneous administration of analgosedation with a neuromuscular blocker (NMB) and is the most common method of establishing definitive airways in emergency settings [2]. The most commonly utilized NMB categories in emergency airway management include depolarizing NMBs (D-NMBs) such as succinylcholine and non-depolarizing NMBs (ND-NMBs) such as rocuronium. While pharmacokinetic and safety profiles differ slightly, both agents are widely considered to be safe choices for general emergency airway management when appropriate forethought to patient specific factors is applied [3,4,5,6,7,8,9,10].

Significant controversy remains, however, regarding the optimal pharmacologic approach for endotracheal intubation in patients suffering from SE [11,12,13,14,15]. While societal guidelines via the American Epilepsy Society, Emergency Neurologic Life Support and Neurocritical Care Society exist to aid clinicians managing SE, it's notable that none of these guidelines address the specific topic of paralytic selection in SE intubations [16,17,18]; as a result, conflicting expert opinions continue to circulate in the setting of limited objective trial data. ND-NMB proponents argue that patients presenting with SE are frequently found down by first responders with varying degrees of prolonged tonic-clonic activity that creates risk of rhabdomyolysis and resultant critical hyperkalemia following administration of D-NMBs [11]. Some patients with SE additionally may have underlying denervating disease (e.g. multiple sclerosis, amyotrophic lateral sclerosis, etc.) which could also increase risk of critical hyperkalemia [19]. D-NMB proponents prefer the rapid offset time of D-NMBs to the long-offset time of ND-NMBs, as cerebral epileptiform activity is not affected by muscular paralysis – it's merely outwardly masked [15].

Evidence supporting D-NMB selection in this setting is sparse but encapsulated as follows: 1) it's known that longer duration of ongoing, uncontrolled seizure activity is associated with permanent neurologic sequelae [20]. 2) NMBs such as succinylcholine / rocuronium facilitate intubation through generalized muscular paralysis but don’t affect cerebral ictal activity – this feature makes them useful adjuncts in electroconvulsive therapy patients to prevent physical harm from convulsive activity [21]. 3) Patients who receive induction dosing of a sedative with anticonvulsant properties such as propofol or ketamine don’t universally achieve cessation of seizure activity - seizures that remain intractable despite 24 hours of general anesthetic infusions constitute a syndrome known as super-refractory SE [22]. D-NMB proponents assimilate this evidence, arguing the 60-minute paralytic period of ND-NMBs is a vulnerable period whereby ictal activity could lose physical correlate which might otherwise prompt additional anticonvulsant medication [14-15] - though real-world data examining this final point has previously been lacking.

The present study seeks to further understand safe airway management in the specific patient population of SE, with particular attention to surrogate functional neurologic outcomes following RSI as a function of D-NMB vs. ND-NMB paralytic selection. While SE is a life-threatening emergency that is one of the most common neurologic syndromes prompting RSI in acute care settings, there are no studies to date specifically evaluating this question via documented, patient centered outcomes; the present study seeks to fill this gap.

## Materials and Methods

The pathophysiological interaction between the variables of interest is illustrated in Figure 1. We hypothesized that D-NMBs, as a result of shorter duration of action, would lead to improved functional neurologic outcomes at hospital discharge via reduced incidence of ongoing masked or “subclinical” seizure activity as a result of a shorter paralysis period – allowing clinicians to up-titrate powerful anticonvulsant anesthetics such as propofol as needed. We additionally speculated that risk of life-threatening hyperkalemia and resultant peri-intubation ventricular or pulseless arrhythmias would be clinically insignificant. Specifically, we posited that muscle fasciculations may pose minimal risk for SE patients given many of these patients are already experiencing full body tonic-clonic contractions prior to induction.

**Fig. 1.**
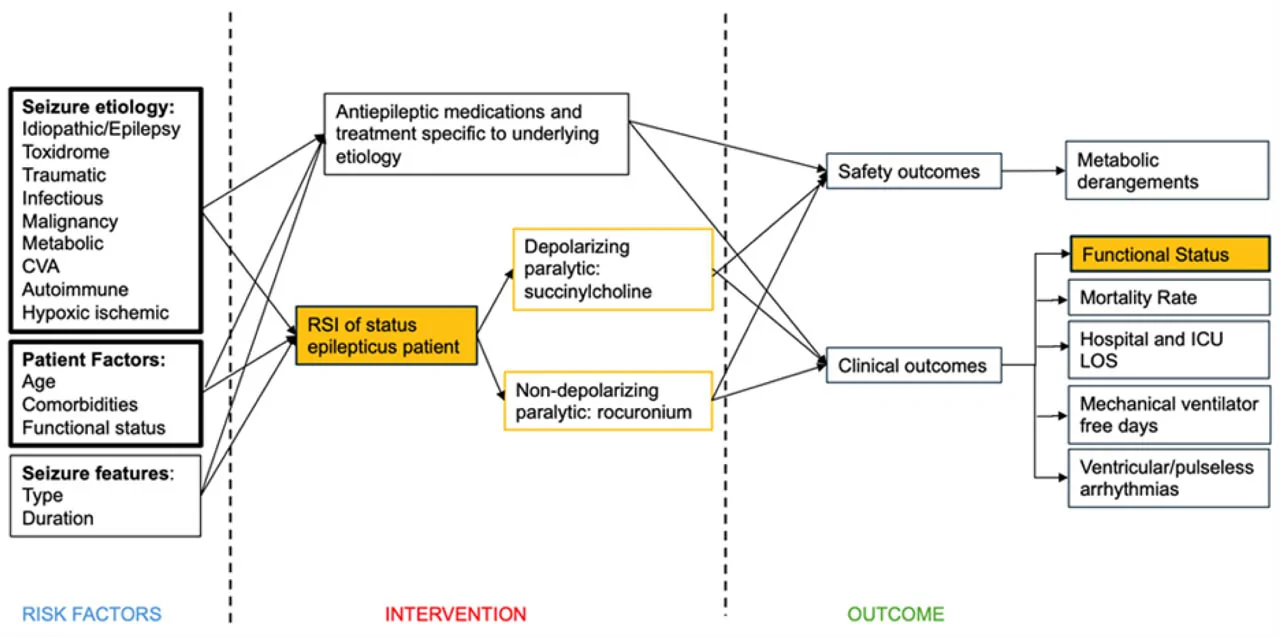
A Focus on RSI Pharmacotherapy in SE Directed acyclic graph depicting complex interactions in SE patients. Orange filled boxes represent study concepts under evaluation. Orange outlined boxes highlight study interventions under evaluation. Bolded, black, outlined boxes represent variables for which patient-level data was available to control in subsequent analyses

The study was approved by the institutional review board (IRB 24-000491). A target trial emulation (TTE) design was utilized, with time of NMB administration designated as time zero and primary outcome of interest as functional neurologic outcomes via the pragmatic surrogate measure “need for escalation in level of care upon hospital discharge.” This approach is supported via previous studies demonstrating strong correlation between “need for escalation in level of care” at discharge and 3-month Modified Rankin Scale (r=0.71) [23-24].

Using a proprietary, institutional dataset (Mayo Data Explorer), adult patients were identified via review of all emergency medicine provider notes on record which included the key search phrase “status epilepticus” in provider documentation. This preliminary search identified 1,455 potential cases between May 2000 and March 2024 at a large, academic hospital system.

Two physicians (P.K and J.G.M) reviewed cases to ensure inclusion criteria were met and categorize seizure etiology into one of 9 possible categories (idiopathic / epilepsy, toxidrome, traumatic brain injury [TBI], central nervous system [CNS] infection, intracranial neoplasm, metabolic derangement, spontaneous intracranial hemorrhage / stroke, autoimmune, and hypoxic / ischemic). A third author (D.G) separately evaluated a random sampling of 50 patients to ensure adequate interrater reliability amongst seizure etiology determinations, and inter-observer agreement of 90% was found following this additional chart review. Across three flagship hospitals in separate states (Minnesota, Florida, Arizona) and numerous affiliated health system hospitals, a total of 480 unique patients intubated utilizing NMBs for suspected SE meeting full study criteria were identified for analysis (Figure 2).

**Fig. 2.**
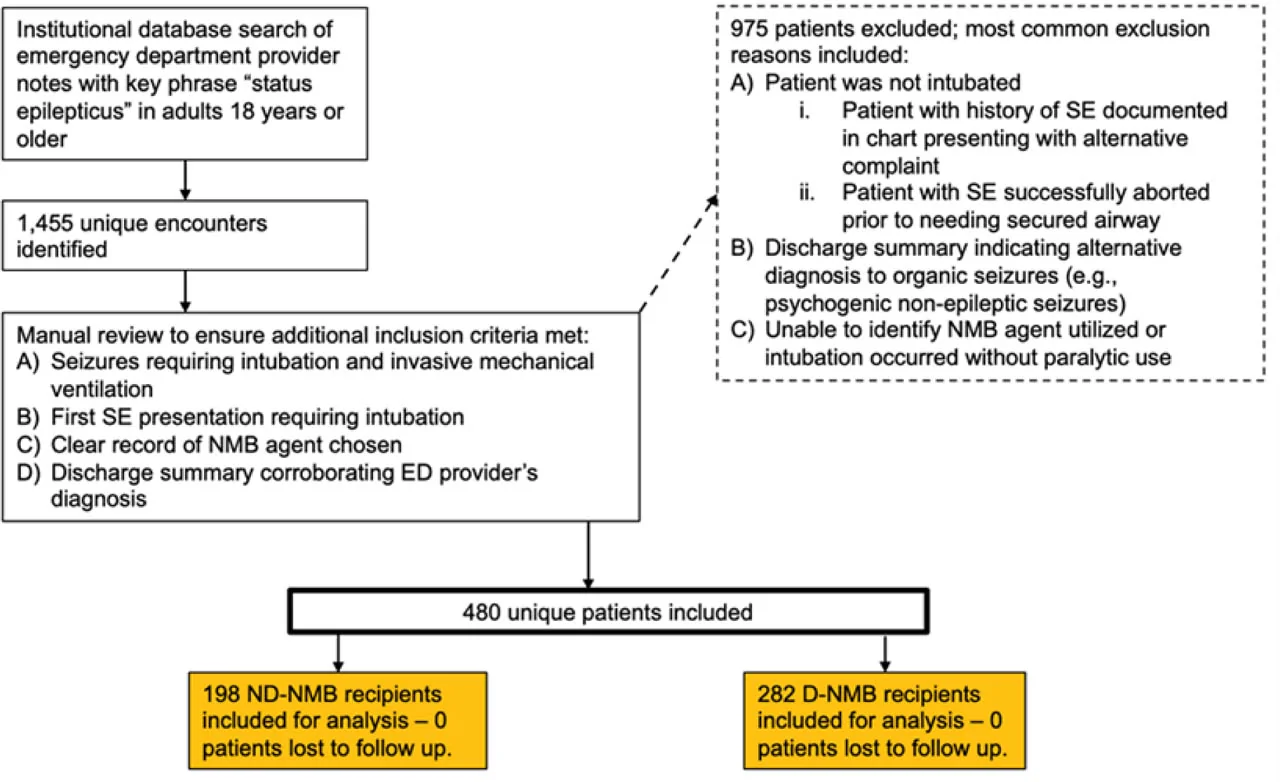
Cohort flow diagram

While generalized convulsive status epilepticus (GCSE) is typically defined as a convulsive seizure whose motor manifestations extend beyond five minutes duration or by multiple convulsive seizures without interictal recovery of consciousness [25], we created an operational definition for research purposes. Specifically, patients in whom the treating emergency physician suspected “status epilepticus” (with encounters identified via database search of this key phrase), with chart documentation of witnessed or reported seizure activity, were considered as potential SE patients. As our study aim was to evaluate the optimal pharmacologic NMB class for intubation, only intubated patients were included. The vast majority of ED documentation noted features consistent with tonic-clonic semiology in our cohort, though at least one patient demonstrated isolated tonic features. Patients profoundly post-ictal in between recurring seizures were included as well as patients actively seizing. This approach ensured logistical feasibility towards eligible patient identification while maintaining evidentiary basis – Zeidan et al. note that, of 98 ICU patients who were admitted to French ICUs after intubation with initial suspicion for GCSE, retrospective adjudication by neurologists confirmed GCSE in 88.8% of cases [26].

An additional layer of chart review of hospital discharge summaries was undertaken to ensure initial ED clinician suspicion for diagnosis of SE was clinically re-confirmed during hospitalization (e.g., patients in whom discharge summaries revealed primary suspicion for psychogenic nonepileptic seizures as underlying diagnosis were excluded). Patients in whom NMBs were not used or in whom the specific NMB utilized was not apparent were excluded from analysis – since the vast majority of patients undergoing emergency intubation receive NMB to facilitate first pass success, our aim was to clarify the optimal agent (ND-NMB or D-NMB).

This study was designed using a TTE framework (Table 1) to approximate the causal contrast that would have been estimated in a pragmatic randomized trial comparing depolarizing versus non-depolarizing neuromuscular blockers during RSI for suspected SE. Eligible participants included adults ≥18 years old presenting with clinically suspected SE requiring endotracheal intubation and documented receipt of a single neuromuscular blocking agent during the index encounter. Time zero was defined as the moment of NMB administration, at which point eligibility assessment, treatment assignment, and follow-up were aligned to minimize immortal time and selection biases. Patients were excluded if neuromuscular blocker choice could not be determined, if both depolarizing and non-depolarizing agents were administered during induction (e.g., a defasciculating dose of ND-NMB prior to D-NMB), or if subsequent chart review suggested an alternative diagnosis such as psychogenic nonepileptic seizures. To emulate randomized treatment assignment, propensity score matching and inverse probability of treatment weighting (IPTW) were used to balance measured baseline confounders between treatment groups prior to outcome estimation. To preserve analytic independence and align with the TTE framework, each patient was included only once, at the first qualifying encounter. Repeated encounters could violate independence assumptions, overrepresent individual patients, and introduce temporal confounding from prior treatments or outcomes.

**Table 1:** Target Trial Specifications

**Target trial component**	**Emulated design**
Eligibility	Adults ≥18 with suspected SE requiring RSI and a single documented NMB use
Treatment strategies	D-NMB vs. ND-NMB at induction
Time zero	NMB administration
Assignment	Clinician-selected; emulated using propensity methods
Follow-up	Hospital discharge and 30 days
Primary outcome	Escalation in level of care at discharge
Causal contrast	ATE^a^ within the matched analytic population. Because treatment assignment was observational, this is interpreted as an adjusted association rather than proof of causality.
Analysis	Propensity matching/IPTW with weighted outcome models

a Average treatment effect (in the matched, weighted analytic population).

### Measurements and Variables

Patient demographic data and comorbidity status were collected using assigned International Classification of Diseases codes, which were subsequently used to calculate the Charlson Comorbidity Index [27]. Patient admission origins were categorized as home vs. others. ED encounters were matched with subsequent ICU admissions for which the following data were collected: severity of illness at admission (as assessed by APACHE III and SOFA scores within the first 24 hours), ICU length of stay (LOS), total duration of invasive mechanical ventilation (IMV), and discharge status. Laboratory variables—including potassium, creatinine, creatine kinase, and lactic acid—were recorded at three time points: at the time of the event, 48 to 72 hours post-event, and at ICU discharge. Additionally, total hospital LOS and discharge disposition were documented.

With respect to defining our primary outcome, “escalations in level of care” at time of hospital discharge, patients who were independent at home pre-admission who needed new home health services (e.g., regularly visiting RN) at discharge or who required a higher level of care such as skilled nursing facility (SNF) upon discharge were considered to have suffered a functional decline. Likewise, transition from SNF to comfort focused care or hospice was deemed an escalation in light of increased debility. In other words, any increased level of resources required to care for the patient on discharge constituted escalation. Secondary clinical outcomes included peri-intubation pulseless or ventricular arrhythmias, mortality rate, hospital and ICU LOS, and hours of mechanical ventilation. Secondary safety outcomes included laboratory variables as above.

### Statistical Methods

To address potential confounding, propensity score-based matching was performed, primarily using a 1:1 ratio. In some cases, 1:3 matching was applied to maximize the use of available control cases. Subsequently, IPTW was applied within the matched sample to further adjust for residual imbalance in covariates, thereby creating a weighted pseudo-population for outcome estimation. Covariate balance between groups was assessed using standardized mean differences (SMDs), with values below 0.2 considered indicative of minimal bias. A graphical summary of covariate balance is included (Supplementary Figure 1). Covariates accounted for in the weighted analysis included: age, sex, origin (home, SNF, etc.), baseline renal disease, potassium at time of event, etiology of idiopathic or toxidrome (which were suspected to carry best prognosis), and Charlson scores. Following IPTW, weighted outcome analyses were performed to compare patients receiving D-NMBs vs. ND-NMBs. Binary outcomes were analyzed using weighted logistic regression models, and results were reported as odds ratios (OR) with 95% confidence intervals (95% CI). Continuous outcomes demonstrated non-normal and right-skewed distributions and were therefore summarized as medians with interquartile ranges (IQR). The primary estimand was the average treatment effect (ATE), defined as the marginal contrast in outcomes that would be expected if all eligible patients in the weighted analytic population had received a depolarizing NMB versus if all had received a non-depolarizing NMB. Because treatment assignment was nonrandomized, this estimand was approximated using propensity-based matching and IPTW to balance measured baseline covariates. Initial weighted group comparisons for continuous variables were performed using weighted non-parametric rank-based testing. However, because rank-based tests do not directly estimate effect magnitude and may be sensitive to pseudo-population weighting approaches, weighted quantile regression models (τ=0.5) were additionally performed to estimate adjusted median differences (β coefficients) with corresponding 95% confidence intervals and p-values.

Supplementary sensitivity analysis was performed for the two least medically confounded etiologies—idiopathic epilepsy and toxidrome. An additional supplemental sensitivity analysis was conducted focusing on patients admitted from home. Statistical significance was determined using a threshold of p<0.05. All statistical analyses were performed using R Studio.

## Results

### Patient Characteristics

Table 2 depicts a demographic breakdown of all 480 study patients. A total of 198 patients received ND-NMBs (189 rocuronium and 9 vecuronium recipients) and 282 received D-NMBs (succinylcholine). Between the two groups, the depolarizing group was roughly 5 years older on average relative to ND-NMB recipients. Additionally, the depolarizing group had a higher burden of baseline comorbidities, as assessed by higher median Charlson scores (5 vs. 3, p=0.04, Table 2). Lactic acid was elevated in both groups of seizure patients, slightly more so amongst the depolarizing group (3.9 vs 3.16, p=0.01, Table 2); taken together, D-NMB pre-weighting characteristics were most consistent with increased illness severity. Supplementary table 1 demonstrates largely similar seizure etiologies between D-NMB and ND-NMB recipients.

**Table 2:** Baseline Demographics

**Variable**	**Non-depolarizing neuromuscular blockers (N=198)**	**Depolarizing neuromuscular blockers (N=282)**	**P**
Age (years)--mean (STD)	51.4±19.43	56.84±18.10	0.002*
Male--no. (%)	121 (61.1%)	155 (55.0%)	0.18
Reside at home--no. (%)	174 (87.9%)	245 (86.9%)	0.75
Initial APACHE III^a^ score–median (IQR)	77.0 (63.0–97.0)	74.0 (59.0–95.0)	0.11
Initial SOFA score^b^--median (IQR)	6.0 (4.0–8.0)	6.0 (4.0–8.0)	0.40
Charlson score^c^--median (IQR)	3.0 (1.0–7.0)	5.0 (2.0–8.0)	0.04*
Initial potassium (mEq/L)--median (IQR)	3.9 (3.5–4.3)	4.0 (3.6–4.4)	0.42
Initial creatinine (mg/dL)--median (IQR)	0.95 (0.77–1.25)	0.90 (0.70–1.20)	0.22
Initial CK (U/L)--median (IQR)	162.0 (90.0–364.0)	157.0 (76.0–353.0)	0.53
Initial lactic acid (mmol/L)--median (IQR)	3.16 (1.60–6.60)	3.90 (2.10–8.75)	0.01*

a Acute physiology and chronic health evaluation – an ICU severity of illness classification system

b Sequential organ failure assessment – an ICU severity of illness classification system

c An index reflecting long term comorbidity burden

### Raw Analysis

Table 3 presents raw results before controlling for confounders. Our primary outcome, escalation in level of care at hospital discharge, was not statistically significantly different across all 480 study patients presenting with SE (27.3% vs. 28.7%, p=0.73). Secondary outcomes such as discharge to home, duration of mechanical ventilation, and survival also showed no difference based on type of paralytic administered (Table 3).

**Table 3:** Unadjusted (Crude) Results, All Seizure Etiologies, All Origins

**Variable**	**Non-depolarizing neuromuscular blockers (N=198)**	**Depolarizing neuromuscular blockers (N=282)**	**P**	**OR (95% CI)**	**Beta (95% CI)**
Escalation in level of care upon hospital discharge–no. (%)	54 (27.3%)	81 (28.7%)	0.73	1.05 (0.78–1.42)	
Discharged to home--no. (%)	143 (72.2%)	201 (71.3%)	0.53	0.99 (0.87–1.12)	
Survival to discharge--no. (%)	181 (91.4%)	249 (88.3%)	0.18	0.97 (0.91–1.03)	
30 Day survival--no. (%)	176 (88.9%)	263 (93.3%)	0.09	1.05 (0.99–1.11)	
Patients with peri-intubation ventricular or pulseless arrhythmias--no. (%)	2 (1.0%)	3 (1.1%)	0.95	1.05 (0.18–6.19)	
Hospitalization length of stay in days--median (IQR)	4.4 (2.3–8.5)	5.1 (3.0–8.1)	0.3		0.65 (−0.32 to 1.64)
ICU Length of stay in days--median (IQR)	2.1 (1.0–3.6)	1.8 (1.0–3.8)	0.5		−0.26 (−0.60 to 0.02)
Hours of invasive mechanical ventilation--median (IQR)	14.4 (0.72–43.44)	12.72 (0.48–30.72)	0.32		−6.24 (−14.4 to 0.48)
Potassium (mEq/L), 48 to 72H from initial event--median (IQR)	3.80 (3.50–4.10)	3.80 (3.60–4.1)	0.65		0.023 (−0.636 to 0.436)
Potassium (mEq/L) at ICU discharge--median (IQR)	3.90 (3.70–4.20)	3.90 (3.60–4.2)	0.95		−0.008 (−0.048 to 0.298)
Creatinine (mg/dL), 48 to 72H from initial event--median (IQR)	0.88 (0.69–1.19)	0.80 (0.60–1.04)	0.09		−0.08 (−0.184 to 0.004)
Creatinine (mg/dL) at ICU discharge--median (IQR)	0.80 (0.66–1.10)	0.80 (0.65–1.0)	0.27		0.00 (−0.059 to 0.059)
CK (U/L), 48 to 72H from initial event--median (IQR)	1244 (707–11267)	1259 (514–5140)	0.8		−227.0 (−1668.85 to 1193.27)
CK (U/L) at ICU discharge--median (IQR)	197 (115.0–882.0)	157.0 (78.0–390.0)	0.12		−40.0 (−130.69 to 34.90)
Lactic acid (mmol/L), 48 to 72H from initial event--median (IQR)	1.20 (1.00–1.5)	1.20 (0.80–2.0)	0.84		0.100 (−0.48 to 0.57)
Lactic acid (mmol/L) at ICU discharge--median (IQR)	1.50 (1.00–2.1)	1.40 (1.10–2.3)	0.45		−0.100 (−0.29 to 0.56)

### Target Trial Emulation Analysis

Table 4 details the results of the adjusted analysis across all 480 patients following propensity matching, inverse probability of treatment weighting and regression; as seen in Supplementary Figure 1, SMDs of weighted covariates (including but not limited to sex, origin, age, comorbidities as encapsulated by Charlson score, amongst others) were less than 0.2 throughout with exception of age which was less than 0.25. D-NMB use was associated with higher odds of escalation in level of care at hospital discharge (OR 1.35, 95% CI 1.01–1.81) and lower odds of discharge to home (OR 0.71, 95% CI 0.54–0.94). No statistically significant differences were observed for survival to discharge, 30-day survival, or peri-intubation ventricular/pulseless arrhythmias. For continuous outcomes, weighted quantile regression analyses demonstrated no statistically significant median differences between groups for hospitalization LOS, ICU LOS, duration of IMV, potassium levels, creatinine levels, creatine kinase levels, or lactate levels after IPTW adjustment. Although several variables demonstrated statistically significant differences in weighted univariate rank-based testing, these associations were attenuated and no longer statistically significant following weighted quantile regression modeling, suggesting that initial observed differences were likely influenced by residual baseline imbalance and distributional differences rather than robust treatment-associated effects.

**Table 4:** Adjusted Results, All Seizure Etiologies, All Origins

**Variable**	*Non-depolarizing neuromuscular blockers (N=351)	*Depolarizing neuromuscular blockers (N=589)	**P**	**OR (95%CI)**	**Beta (95%CI)**	**Weighted Quantile Regression P**
Escalation in level of care at hospital discharge–no. (%)	85 (24.4%)	176 (30.4%)	0.04*	1.35 (1.01–1.81)*		
Discharged to home--no. (%)	244 (69.6%)	364 (61.7%)	0.02*	0.71 (0.54–0.94)*		
Survival to discharge--no. (%)	330 (95.3%)	539 (95.2%)	0.92	0.98 (0.53–1.80)		
30 Day survival--no. (%)	316 (89.9%)	549 (93.1%)	0.06	1.51 (0.98–2.32)		
Patients with peri-intubation ventricular or pulseless arrhythmias--no. (%)	4 (0.8%)	7 (0.6%)	0.64	0.82 (0.24–2.83)		
Hospitalization length of stay in days--median (IQR)	4.2 (2.3–8.3)	5.2 (3.0–8.3)	0.043*		0.95 (−0.135 to 1.812)	0.126
ICU Length of stay in days--median (IQR)	2.0 (1.0–3.6)	1.8 (1.0–3.8)	0.52		−0.20 (−0.45 to 0.05)	0.267
Hours of invasive mechanical ventilation--median (IQR)	20.88 (9.84–45.84)	18.72 (6.96–42.72)	0.15		−2.18 (−7.15 to 3.04)	0.445
Potassium (mEq/L), 48 to 72H from initial event--median (IQR)	3.9(3.5–4.10)	3.8(3.6–4.10)	0.5		−0.10 (−0.10 to 0.34)	0.187
Potassium (mEq/L) at ICU discharge--median (IQR)	3.9(3.7–4.2)	3.9(3.6–4.2)	0.89		0.00 (0.00 to 0.39)	0.97
Creatinine (mg/dL), 48 to 72H from initial event--median (IQR)	0.88 (0.69–1.19)	0.80 (0.64–1.00)	0.01		−0.08 (−0.18 to 0.02)	0.147
Creatinine (mg/dL) at ICU discharge--median (IQR)	0.80 (0.66–1.10)	0.80 (0.65–1.0)	0.12		0.00 (−0.07 to 0.07)	0.99
Creatine kinase (U/L), 48 to 72H from initial event--median (IQR)	1477 (707–13085)	928 (492–2820)	0.67		−549 (−1682.99 to 1072.28)	0.868
Creatine kinase (U/L) at ICU discharge--median (IQR)	222 (115–948)	147 (76–390)	0.01		−75 (−144.18 to 11.18)	0.225
Lactic acid (mmol/L), 48 to 72H from initial event--median (IQR)	1.2 (1.0–1.8)	1.7 (0.8–2.2)	0.12		0.50 (−0.23 to 0.72)	0.272
Lactic acid (mmol/L) at ICU discharge--median (IQR)	1.5 (1.0–1.9)	1.50 (1.1–2.3)	0.19		0.00 (−0.37 to 0.43)	0.955

* Values are weighted counts and weighted percentages after IPTW; original unweighted group sizes were 198 ND-NMB and 282 D-NMB.

## Discussion

Across 480 suspected SE patients with initially undifferentiated seizure etiologies, ND-NMB receipt was associated with lower adjusted odds of escalation in level of care at discharge compared to D-NMB recipients. This highlights a potential association with improved functional neurologic outcomes, as “need for escalation in level of care” is established as a strong correlate toward validated measures of functional neurologic outcome after acute neurologic injury (association with both 3-month and 12-month modified Rankin) [23-24]. A more guarded interpretation of study results, when considering potential hidden confounders discussed below, might be that the difference between selected agents is negligible, though both interpretations stand in contrast to D-NMB proponent assertion that succinylcholine drastically improves outcomes by facilitating rapid neurologic re-examination [14-15]. With respect to hyperkalemia risk, there was no difference between drug groups when considering important peri-intubation cardiopulmonary events such as ventricular or pulseless arrhythmias (Table 4). On subgroup analysis of patients presenting from home, there was a slight increased risk of 30-day mortality amongst ND-NMB recipients, though the favorable effect size with respect to need for escalation in level of care at discharge was more notable, with 31.4% of D-NMB recipients needing escalation in resource intensity upon discharge relative to only 24.8% of ND-NMBs recipients as seen in Supplementary Table 2 (p=0.04).

While numerous societal guidelines exist to aid clinicians managing SE [16,17,18], none of these major guidelines address nor reference trials examining the topic of NMB selection in SE airway management (a commonly required intervention for this acute neurologic emergency). To our knowledge, the present study is the first to specifically examine real-world, patient centered outcomes with regards to SE airway management.

Though a mechanism whereby D-NMBs might facilitate improved neurologic outcomes has been theorized [15], no obvious mechanism has been speculated for ND-NMBs. One consideration is whether operators utilizing ND-NMBs account for the drug's pitfall of longer duration of action by more aggressively dosing their propofol infusions following intubation, though our study did not collect this data a priori. Conversely, brief resolution of visible convulsions after D-NMB use may provide false reassurance despite ongoing electroencephalographic seizure activity, a recognized phenomenon after apparent clinical control of convulsive SE [28]. This hypothesis requires prospective testing.

### Strengths and Limitations

The discrepancy between the crude and adjusted primary outcome estimates is central to interpretation. In the unadjusted cohort, escalation in level of care did not differ by NMB class (27.3% with ND-NMBs vs. 28.7% with D-NMBs; p=0.73). After propensity-based matching and IPTW adjustment, D-NMB use was associated with higher odds of escalation in level of care (30.4% vs. 24.4%; OR 1.35, 95% CI 1.01–1.81). This shift indicates that the primary finding is materially dependent on adjustment for measured covariates and should be interpreted as an adjusted association rather than evidence of causality [29-30]. The change in estimate likely reflects baseline imbalance and model-based reweighting of measured covariates; however, residual confounding remains possible, particularly from nonrandom clinician-dependent paralytic selection, unmeasured illness severity, seizure duration, sedation strategy, EEG availability, and calendar time. Accordingly, these findings should be considered hypothesis-generating and require prospective confirmation.

Our study carries with it the inherent limitations of retrospective study design-paralytic selection was non-randomized, guided by operator preference, and could have been influenced by unmeasured illness severity. Notably, however, D-NMB recipients were significantly older with greater comorbidity burden; propensity score matching followed by IPTW would therefore be expected to shift the estimate in their favor by neutralizing this baseline disadvantage. Instead, the adjusted analysis favored ND-NMBs, suggesting the observed association is less likely to be an artifact of model over-correction. Directed acyclic graphs were used to identify confounders and colliders, and a target trial emulation methodology was employed to approximate key design features of a pragmatic randomized trial while recognizing that residual and unmeasured confounding remain possible [34].

With respect to potential confounders, considerations include temporal, physician-specific, patient-specific, and seizure-specific confounders. The 24-year inclusion period introduces an important risk of temporal confounding. Airway management, EEG availability, ICU sedation practices, discharge disposition norms, and paralytic preference likely evolved during the study period. In our cohort, NMB class varied significantly by calendar year, and year of admission was not included in the primary propensity model. Therefore, secular trends may have contributed to the adjusted association observed in the primary analysis. This remains an important limitation and supports interpretation of the findings as hypothesis-generating rather than causal. However, the majority of cohort patients presented from 2018 to 2024, a period during which paralytic selection patterns were relatively stable (Supplementary Figure 2), limiting the practical impact of this concern.

Physician-specific confounders of concern include difficult-to-quantify variables which are challenging to directly control (e.g., thresholds for intubation, agent preferences, etc.). Certainly, these are important, as secondary analysis of the nation-wide, multi-center ESSETT Trial showed strong site-specific variability in intubation rates of SE patients (4-32% of pediatric SE patients, with decreased variability of 19–39% among adult SE patients) [32] – by focusing on adult patients with a robust sample size in a single, large health system, this has been further minimized.

In considering patient-specific confounders, baseline level of debility (originating from either home vs. SNF), age, and overall comorbidity burden (Charlson score) were adjusted for as covariates in our model. To further control seizure specific confounders (e.g., metastatic cancer with intracranial involvement), sensitivity analysis was facilitated via study author categorization of cases into nine possible seizure etiologies. As we considered them least likely to suffer from extensive comorbid conditions, we devoted additional sensitivity analysis in Supplementary Table 4 to idiopathic epilepsy and toxidrome SE patients presenting from home. Even in this carefully selected subgroup, the theorized neurologic advantages of D-NMBs mediated by more rapid clinical re-examination were not seen, with ND-NMBs offering at least similar functional neurologic outcomes as demonstrated by lack of difference in need for escalation of level of care at discharge.

When considering the reasonableness of our chosen primary surrogate outcome, certainly an optimal, prospective, randomized controlled trial would assess functional neurologic outcomes directly via validated clinical scoring tools such as Glasgow Outcome Scale or Modified Rankin Scale. However, it's known that attempts to adjudicate these scores retrospectively via chart review are inaccurate and unreliable [31]. Given a lack of feasibility in utilizing direct measures, an objective, strong correlate towards 3-month Modified Rankin was chosen as the primary surrogate for functional neurologic outcome (“need for escalation in level of care” upon hospital discharge) (r=0.71) [23-24].

Additionally, the chosen surrogate of “need for escalation in level of care” could capture non-neurologic reasons for new SNF placement, new hospice referrals, or other escalations - on review of discharge summaries, it was clear some patients had medically complicated hospital courses, with shock, respiratory failure, and other acute processes potentially contributing to deconditioning with resultant new SNF needs on discharge rather than cognitive decline alone. This is not unusual in critically ill patients.

In considering potential for misclassification biases, a particular study strength included physician author chart review of all 480 patients included in our study. As correct identification of SE was deemed critical, each emergency physician encounter was examined to ensure: 1) the key search phrase “status epilepticus” was of relevance to the current rather than historical encounters, 2) witnessed or reported seizure like activity was documented, and 3) associated hospital discharge summaries corroborated initial emergency physician suspicion for SE (e.g., psychogenic seizure cases were excluded).

The main determinant of prognosis following SE is etiology [11]; while significant measures outlined above were taken to control for this, additional SE outcome determinants such as specific seizure duration precisely quantified via continuous EEG (cEEG), exact AED regimen dosing, and additional important yet granular details will best be captured via future prospective, randomized controlled trials that might address this question.

An additional noteworthy strength of the study is the large sample size of almost 480 patients, as this likely led to improved balancing between the two groups with respect to non-measured variables such as anticonvulsant regimen. As all patients are from a single health system, we would expect increased uniformity in practice patterns with respect to the decision to intubate as well as optimal anticonvulsant regimen selection, which is typically benzodiazepines, a second line antiepileptic drug such as fosphenytoin/levetiracetam/valproate (as per the large, nation-wide ESSETT trial which showed similar efficacy between all three), and an anesthetic infusion with anticonvulsant properties such as propofol [33].

Finally, and of note, our study likely included a broad variety of SE severity. It is possible that different results might be observed with specific focus upon more severe SE disease states such as patients presenting with “super-refractory SE,” defined as a persistent state of SE 24 hours following onset of anesthetic intravenous treatment [22]. With this in mind, authors agree with expert recommendations to consider cEEG monitoring or reversal with sugammadex if ND-NMBs are utilized [11].

## Figures and Tables

**Supplementary Table 1:** Seizure Etiology Demographics

**Seizure Etiology**	**Non-depolarizing NMB**	**Depolarizing NMB**	**Total**
Idiopathic / Epilepsy--no. (%)	107 (54%)	160 (56.7%)	267 (55.6%)
Toxidrome--no. (%)	30 (15%)	29 (10.3%)	59 (12.3%)
Traumatic Brain Injury--no. (%)	4 (2%)	7 (2.5%)	11 (2.3%)
CNS Infection--no. (%)	5 (2.5%)	8 (2.8%)	13 (2.7%)
Intracranial Neoplasm--no. (%)	17 (8.6%)	40 (14.2%)	57 (11.9%)
Metabolic Derangement--no. (%)	16 (8.1%)	17 (6%)	33 (6.9%)
Spontaneous Intracranial Hemorrhage / Stroke--no. (%)	15 (7.6%)	17 (6%)	32 (6.7%)
Autoimmune--no. (%)	3 (1.5%)	2 (0.7%)	5 (1%)
Hypoxic-Ischemic--no. (%)	1 (0.5%)	2 (0.7%)	3 (0.6%)
Total	198	282	480

**Supplementary Table 2:** Adjusted Results, Patients From Home

**Variable**	**Non-depolarizing neuromuscular blockers (N=310)**	**Depolarizing neuromuscular blockers (N=509)**	**P**
Escalation in level of care at hospital discharge–no. (%)	77 (24.8%)	160 (31.36%)	0.046
Discharged to home--no. (%)	244 (78.7%)	364 (71.5%)	0.022
Survival to discharge--no. (%)	295 (96.6%)	469 (95.7%)	0.629
30 Day survival--no. (%)	282 (90.8%)	479 (94.2%)	0.050
Patients with peri-intubation ventricular or pulseless arrhythmias--no. (%)	1 (0.5%)	5 (0.9%)	0.283
Hospitalization length of stay in hours--median (IQR)	91.62 (52.56–184.38)	119.52 (71.28–194.88)	0.018
ICU Length of stay in hours--median (IQR)	47.28 (22.08–78.72)	42.48 (23.04–86.16)	0.611
Hours of invasive mechanical ventilation--median (IQR)	19.92 (9.36–43.2)	18.24 (6.24–38.88)	0.243
Potassium, 48 to 72H from initial event--median (IQR)	3.9(3.6–4.1)	3.8(3.6–4.1)	0.880
Potassium at ICU discharge--median (IQR)	3.9(3.7–4.2)	3.9(3.6–4.2)	0.739
Creatinine, 48 to 72H from initial event--median (IQR)	0.88 (0.69–1.30)	0.80 (0.66–1.00)	0.011
Creatinine at ICU discharge--median (IQR)	0.81 (0.66–1.18)	0.80 (0.67–1.03)	0.237
Creatine kinase, 48 to 72H from initial event--median (IQR)	1477 (780–11267)	928 (492–2820)	0.590
Creatine kinase at ICU discharge--median (IQR)	222 (118–882)	166 (82–415)	0.095
Lactic acid, 48 to 72H from initial event--median (IQR)	1.1 (1.1–1.8)	1.7 (0.2–2.2)	0.329
Lactic acid at ICU discharge--median (IQR)	1.5 (1.1–2.00)	1.50 (1.0–2.3)	0.620

**Supplementary Table 3:** Baseline Demographics, Seizures Due to Idiopathic Epilepsy or Toxidrome Presenting from Home

**Variable**	**Non-depolarizing neuromuscular (N=116)**	**Depolarizing neuromuscular blockers (N=158)**	**P**
Age--mean (STD)	48.37±18.66	54.91±18.61	0.004
Male--no. (%)	74 (63.8%)	90 (57.0%)	0.254
Initial APACHE III score--median (IQR)	77.0 (64.0–93.0)	73.50 (56.5–88.0)	0.128
Initial SOFA score--median (IQR)	6 (4–8)	6 (4–8)	0.854
Charlson score--median (IQR)	2.0 (1.0–6.0)	4.00 (1.00–7.00)	0.004
Initial potassium--median (IQR)	3.80 (3.50–4.10)	3.90 (3.60–4.40)	0.067
Initial creatinine--median (IQR)	0.95 (0.76–1.20)	0.95 (0.70–1.11)	0.423
Initial CK--median (IQR)	171.0 (109.0–382.0)	220.0 (113.0–395.0)	0.829
Initial lactic acid--median (IQR)	3.10 (1.60–7.30)	3.75 (2.07–8.41)	0.130

**Supplementary Table 4:** Adjusted Results, Seizures Due to Idiopathic Epilepsy or Toxidrome Presenting from Home

**Variable**	**Non-depolarizing neuromuscular (N=191)**	**Depolarizing neuromuscular blockers (N=305)**	**P**
Escalation in level of care--no. (%)	38 (20.0%)	57 (18.4%)	0.611
Discharged to home--no. (%)	161 (84.5%)	255 (83.8%)	0.840
Survival to discharge--no. (%)	187 (98.1%)	302 (99.2%)	0.308
30 Day survival--no. (%)	183 (95.8%)	299 (98.1%)	0.146
Patients with peri-intubation ventricular or pulseless arrhythmias--no. (%)	2 (0.8%)	2 (0.6%)	0.635
Hospitalization length of stay in hours--median (IQR)	92.64 (49.92–183.12)	114.1 (68.16–174.24)	0.033
ICU Length of stay in hours--median (IQR)	47.28 (23.04–83.04)	41.6 (21.17–70.28)	0.176
Hours of invasive mechanical ventilation--median (IQR)	20.88 (9.36–39.36)	18.12 (7.14–37.22)	0.273
Potassium, 48 to 72H from initial event--median (IQR)	3.7(3.5–3.9)	3.8(3.5–4.0)	0.468
Potassium at ICU discharge--median (IQR)	3.9(3.7–4.1)	3.9(3.6–4.1)	0.638
Creatinine, 48 to 72H from initial event--median (IQR)	0.9 (0.69–1.19)	0.76 (0.60–0.95)	<0.001
Creatinine at ICU discharge--median (IQR)	0.83 (0.70–1.10)	0.80 (0.65–1.0)	0.043
Creatine kinase, 48 to 72H from initial event--median (IQR)	1037 (622–2541)	928 (536–2820)	0.791
Creatine kinase at ICU discharge--median (IQR)	234 (128–944)	157 (112–466)	0.028
Lactic acid, 48 to 72H from initial event--median (IQR)	1.1 (1.1–2.3)	1.0 (0.7–2.2)	0.498
Lactic acid at ICU discharge--median (IQR)	1.4 (1.10–2.0)	1.50 (1.1–2.3)	0.232

**Supplementary Figure 1:**
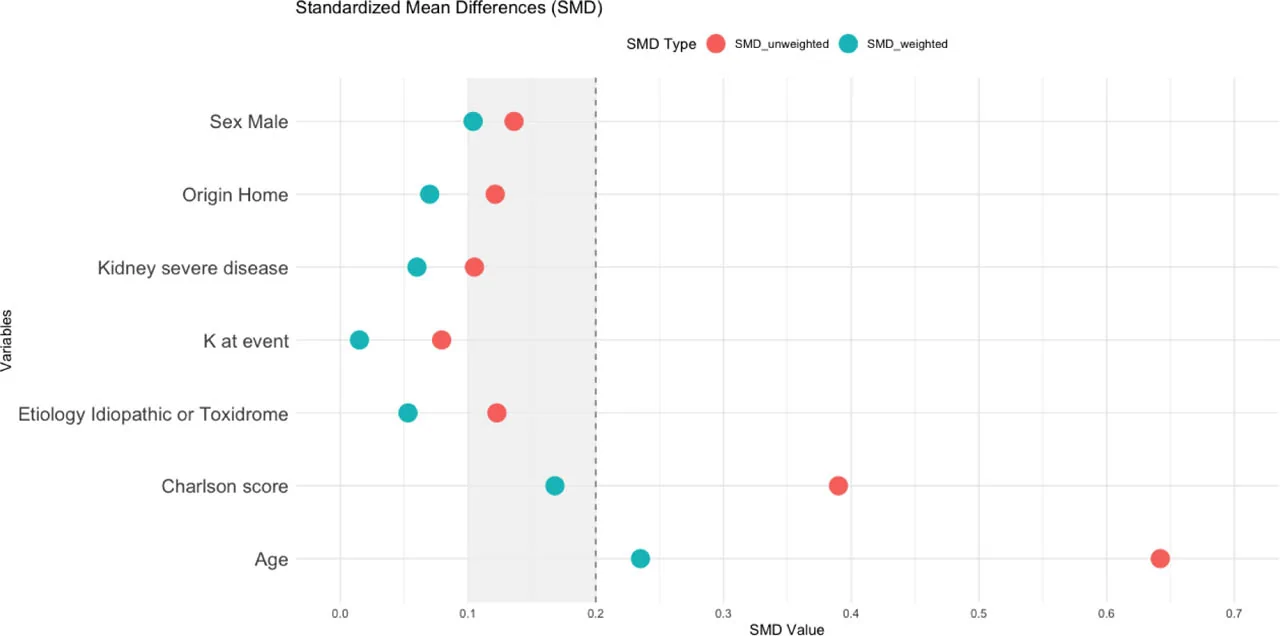
Standardized Mean Differences (SMDs)

**Supplementary Figure 2:**
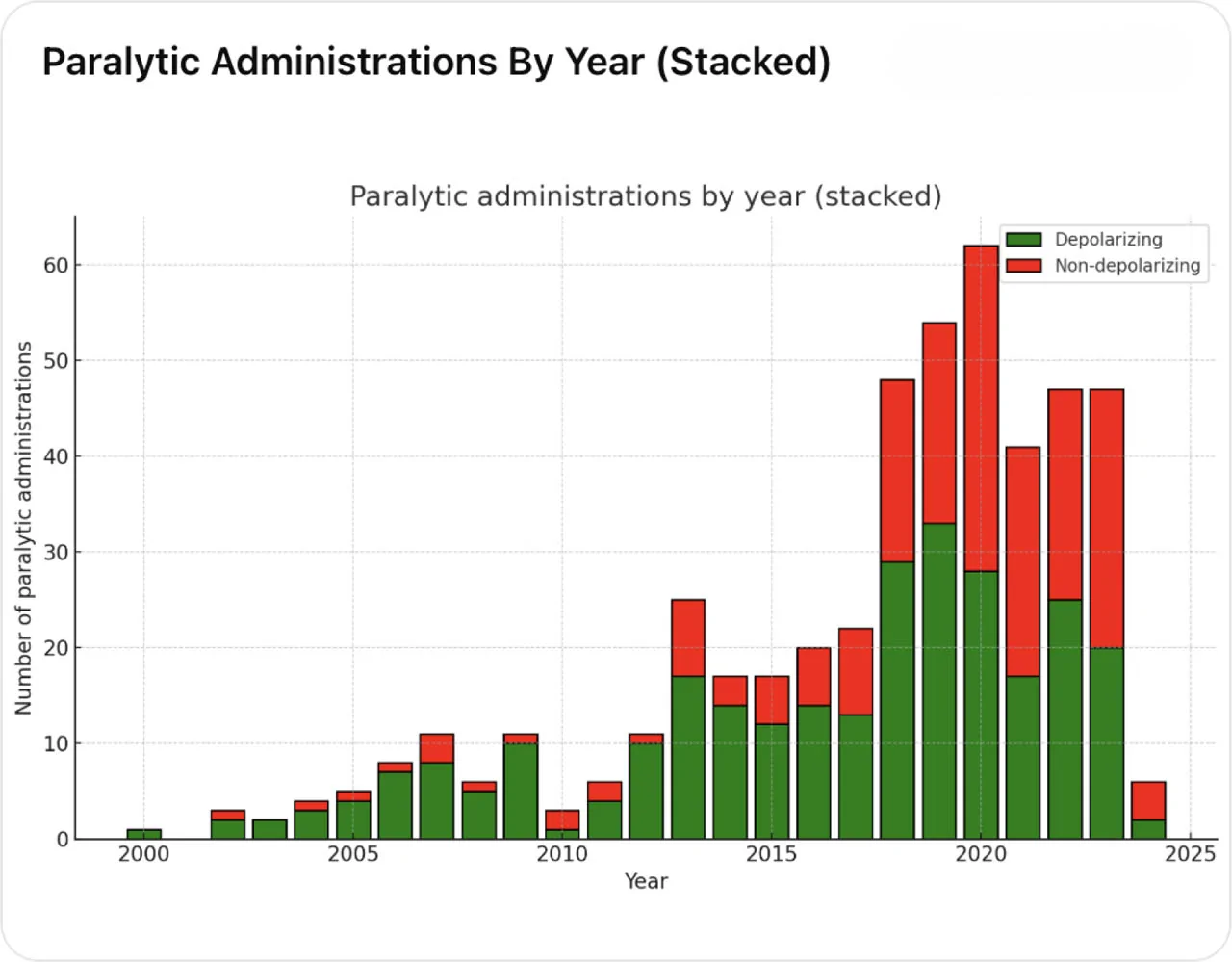
Trend of use of neuromuscular blocking agents by years Chi-square across years: χ^2^ = 43.12 (df = 23), p = 0.0067 → distribution varies by year. Logistic trend (Depolarizing ~ year): OR per +1 year = 0.891 (95% CI 0.852–0.931), p = 2.35e-07 → significant shift away from depolarizing over time.
